# Current status and influencing factors of health literacy among older adults in combined medical and nursing care institutions: a cross-sectional study

**DOI:** 10.3389/fpubh.2023.1323335

**Published:** 2024-01-16

**Authors:** Simin Tao, Silu Sun, Shiying Wu, Tao Peng, Li Cao, Min Yan, Jie Ma, Hui Li

**Affiliations:** ^1^School of Nursing, Chengdu Medical College, Chengdu, China; ^2^Hospital Office, Chengdu Elderly Care Hospital, Chengdu, China; ^3^Sichuan Yibeikang Technology Co., LTD, Chengdu, China; ^4^Department of Geriatrics, West China Hospital, Sichuan University, Chengdu, China

**Keywords:** combined medical and nursing care, nursing care institutions, older adults, health literacy, influencing factors

## Abstract

**Introduction:**

Health literacy is linked to the health status of older adults and is a critical component in achieving active aging. This study to characterize the current health literacy status among older adults in combined medical and nursing care institutions, as well as the factors that influenced this status.

**Methods:**

This cross-sectional study used a convenience sampling method to select 740 older adults who were in 16 combined medical and nursing care institutions in Chengdu, China, from July 2022 to June 2023, using the Health Literacy Management Scale, the Social Support Rating Scale, and the Short Form-36 Health Survey.

**Results:**

The results showed a health literacy score of 75.25 ± 12.33, the percentage of older adults with basic health literacy was 6.33%, a social support score of 37.37 ± 6.10, and a health-related quality of life score of 55.34 ± 15.48. Factors influencing health literacy included age, educational level, previous occupation, family size, monthly per capita household income, and health-related quality of life.

**Discussion:**

Health literacy is an essential factor in improving the health status of older adults. Relevant departments should therefore improve health education and health promotion interventions for older adults in combined medical and nursing care institutions to improve health literacy.

## 1 Introduction

According to China's National Bureau of Statistics, at the end of 2021, the number of people in China over 60 years of age was 267 million, accounting for 18.9% of the population (1). It is expected that around 2035, China's older adults population ≥60 years of age will exceed 400 million, entering a demographic stage with a large percentage of older adults (2). China's per capita healthy life expectancy in 2021 was 78.2 years (3). However, older adults generally suffer from a variety of chronic diseases. In China, there are more than 40 million disabled and semi-disabled older adults (4), so their health condition is not optimistic. Their demand for health services is urgent and imposes a heavy burden on medical and healthcare systems. The 14th Five-Year Plan for the Health of the older adults (5) states that strengthening health education for older adults and improving their health literacy were the primary goals for their health over the past 5 years.

The main ways for older adults in China to age are in communities or institutions. However, with the aging of China's population, the traditional family model of old age care is decreasing, and institutional care is gradually becoming an essential part of the old age care model (6). As most older adults in nursing care institutions have a lower level of education, declining physical function, a heavier burden of disease, and a poorer ability to perform their daily life tasks, there has been a significant increase in the demand for healthcare services for older adults in nursing care institutions, in addition to their material needs.

Health literacy is the ability of individuals to access, process, and understand basic health information and services, and to use them to make appropriate healthcare decisions (7, 8). The results of the 2020 report on monitoring the health literacy of Chinese residents showed that the health literacy level of older adults in the 65–69 year age group was 8.49%, which was the lowest level, when compared with other age groups (9). Compared with the health literacy level of 5.60% in the 65–69 year age group in the 2012 Health Literacy Monitoring Report of Chinese Residents (10), the health literacy level of older adults has shown a steady improvement. The “Healthy China 2030” plan states that health literacy levels should be raised through health education and the promotion of healthy lifestyles (11). The Chinese Government has also stated that health literacy is an essential determinant of health. Improving health literacy is therefore one of the most fundamental, cost-effective, and efficient measures for upgrading health.

Older adults are a vulnerable group when considering health literacy (12, 13). Inadequate health literacy will lead to an increase in the length of hospitalization, higher healthcare expenditures, lower utilization of health services, and poorer adherence to healthcare, which in turn will affect the physical health of older adults (14, 15). In particular, for older adults living in nursing care institutions, the living environment is more closed, with less access to external information, and most of them suffer from chronic diseases (16) and lack appropriate health knowledge and information. There is therefore an urgent need to improve their health literacy to achieve positive and healthy aging. Health literacy is a strong predictor of health status in older adults (17, 18). From an individual perspective, improved health literacy enables older adults to better self-manage their health, and thus improve their health and health-related quality of life (19); and from a socio-economic perspective, improved health literacy partially reduces the pressure and financial burden on the National Health Service (20).

Several studies have reported that health literacy significantly influences health outcomes, and has a crucial role in an individual's physical and mental health (16, 21). Individuals with lower health literacy devote less attention to their health status and are more inclined to have unhealthy behaviors, which in turn leads to a lower quality of life (22). In addition, some studies have shown that social support is an influential factor linking health literacy and quality of life. Good social support enhances an individual's health-promoting behaviors and improves their quality of life (23).

The purpose of this study was therefore to investigate the health literacy of older adults in combined medical and nursing care institutions, and to identify influential factors, to provide a reference basis for the subsequent development of health literacy intervention strategies, and to improve the overall health-related quality of life of the older adults in combined medical and nursing care institutions.

## 2 Methods

### 2.1 Participation and data collection

This was a cross-sectional study, and a convenience sampling method was used to select older adults in 16 combined medical and nursing care institutions in Chengdu City from July 2022 to June 2023. Inclusion criteria included the following: age ≥ 60 years, length of stay ≥90 days, clear consciousness and regular language expression, and informed consent. Exclusion criteria included the following: a cognitive or mental disorder, and a visual or hearing impairment. The sample size was calculated using the following formula (24)$:Z_{1-\alpha /2}^{2}P\left(1-P\right)/\delta^{2}$. According to the 2020 report on monitoring the health literacy of Chinese residents, the health literacy level of Chinese seniors was 8.49%. Therefore, this study set *P* = 0.0849, α = 0.05, *Z*_1−α/2_ = 1.96, and δ = 0.03. Based on this formula, the sample size was calculated to be 332, and the minimum sample size was estimated to be 399, considering the 20% sample non-response percentage. To make the results more representative, 740 questionnaires were distributed, 13 questionnaires with incomplete information was excluded, and 727 valid questionnaires (98.24%) were recovered for analysis.

This study used a questionnaire survey method conducted on-site by uniformly trained investigators using a one-on-one approach. Prior to the survey, the purpose and content of the study were explained to older adults, and the questionnaire was independently completed by the older adults. Those who were unable to complete the questionnaire had the questionnaire entries read by the investigator, who completed the questionnaire on their behalf, based on their responses. The survey took 15–20 min to complete, and all questionnaires were collected immediately and checked for completeness. This study was approved by the Chengdu Medical College Ethics Committee (CMCEC-2022NO.42). The participants provided their written informed consent to participate in the study.

### 2.2 Measures

#### 2.2.1 General information

The data included was designed by the investigators, and included sex, age, marital status, educational level, previous occupation, family size, monthly per capita household income, smoking status, and alcohol consumption.

#### 2.2.2 Health literacy

There is currently no general health literacy scale for older adults in China (25). Due to the prevalence of chronic diseases among older adults in China, this study used the Health Literacy Management Scale to measure the health literacy level of older adults in nursing care institutions. The scale was compiled by Haolin Sun (26), including four dimensions of ability to access information, ability to communicate and interact, willingness to improve health, and willingness to support financially, with a total of 24 entries, using a five-level Likert scale, with scores from 1 to 5 representing “very difficult” to “not difficult at all,” and a total score of 24–120, with a higher score indicating a higher level of health literacy. A questionnaire score of 80% or more of the total score was considered to have basic health literacy, and its number as a percentage of the total number of people surveyed was the level of health literacy. The Cronbach α coefficient for this scale was 0.908.

#### 2.2.3 Social support

The Social Support Rating Scale was designed by Xiao (27), including three dimensions of objective social support, subjective social support, and availability of social support, with a total of 10 entries and a total score of 12–66 points, with higher scores indicating higher levels of social support. The Cronbach α coefficient for this scale study was 0.765.

#### 2.2.4 Health-related quality of life

Short Form-36 Health Survey, the Quality of Survival Evaluation Scale or the Health Survey Short Form (28), is a measurement scale developed by the Bureau of Medicine Research Group. The Chinese version of the scale (29) consisted of a reported health transition, eight dimensions of physical functioning, physical role, bodily pain, general health, vitality, social functioning, emotional role, and mental health, totaling 36 entries. According to the weight of each entry, the sum of the scores of each entry in each dimension was separately calculated, and the crude score of each dimension was calculated. Finally, the crude score was converted to a standard score from 0 to 100. The conversion formula was the following: standard score = (actual score – lowest score of the entry)/(highest score of the entry – lowest score of the entry) × 100. The standardized score ranged from 0 to 100, with higher scores representing a better health-related quality of life. The Cronbach α coefficient for this scale was 0.852.

### 2.3 Statistical analysis

Data were entered using Epidata 3.1 software and statistically analyzed using SPSS 26.0 statistical software for Windows (SPSS, Chicago, IL, USA). Quantitative information was expressed as ($\bar{x}$ ± S) for normal distributions and *M* (P_25_, P_75_) for parametric distributions, and count information was expressed as the frequency and percentage. One-way analysis was performed using the independent samples *t*-test and one-way analysis of variance. The relationships between health literacy, social support, and health-related quality of life were determined using Pearson's correlation analysis. A *multivariate* stepwise linear regression analysis was conducted with variables that were meaningful in the univariate analysis as independent variables, to identify factors influencing the health literacy of older adults in combined medical and nursing care institutions. The test level α was set at 0.05 unless otherwise stated.

## 3 Results

### 3.1 Health literacy, social support, and health-related quality of life scores for older adults in combined medical and nursing care institutions

The results of this study showed that the health literacy scores of older adults in combined medical and nursing care institutions was 75.25 ± 12.33, the percentage of older adults with basic health literacy was 6.33%, and the social support score was 37.37 ± 6.10. The health-related quality of life score was 55.34 ± 15.48. The scores of each dimension of the scale are listed in Table 1.

**Table 1:** Health literacy, social support, and health-related quality of life scores for older adults in combined medical and nursing care institutions.

**Scale/Subscale**	**Scores**	**Minimum**	**Maximum**
Health literacy total scales	75.25 ± 12.33	41	120
**Subscales**			
Ability to access information	25 (21, 31)	9	45
Ability to communicate and interact	29 (27, 32)	16	45
Willingness to improve health	14 (12, 15)	6	43
Willingness to support financially	6 (6, 8)	2	10
Social support rating total scale	37.37 ± 6.10	19	52
**Subscales**			
Objective social support	10 (8, 11)	3	16
Subjective social support	21 (18, 23)	12	29
Availability of social support	7 (5, 9)	3	12
Health-related quality of life total scales	55.34 ± 15.48	5.56	96.13
**Subscales**			
Physical functioning	60 (35, 75)	0	100
Physical role	0 (0, 25)	0	100
Bodily pain	62 (52, 74)	12	100
General health	45 (35, 55)	0	92
Vitality	70 (60, 80)	0	100
Social functioning	62.5 (50, 75)	0	100
Emotional role	100 (0, 100)	0	100
Mental health	72 (68, 80)	0	92
Health transition	25 (25, 50)	0	100

### 3.2 A one-factor analysis of health literacy scores of older adults in combined medical and nursing care institutions

The univariate analysis of this study showed that there were statistically significant differences between the nine variables of different sexes, age, marital status, educational levels, previous occupation, family size, monthly per capita household income, smoking status, and alcohol consumption, when compared to health literacy scores (*P* < 0.05), as shown in Table 2.

**Table 2:** A one-factor analysis of health literacy scores of older adults in combined medical and nursing care institutions.

**Variable**	***N***	**Percentage (%)**	**Scores**	**Being health literate (%)^a^**	**Statistical value**	***P*-value**
**Sex**						
Male	345	47.5	77.01 ± 11.43	21 (6.09)	*t* = 3.688	<0.001
Female	382	52.5	73.66 ± 12.91	25 (6.54)		
**Age (years)**						
60–69	263	36.2	78.89 ± 12.92	27 (10.27)	*F* = 17.717	<0.001
70–79	320	44.0	74.33 ± 11.04	15 (4.69)		
80–89	134	18.4	71.27 ± 12.11	4 (2.99)		
≥90	10	1.4	62.30 ± 9.04	0 (0.00)		
**Marital status**						
Married	613	84.3	76.18 ± 12.15	43 (7.01)	*F* = 8.667	<0.001
Divorcee	2	0.3	85.00 ± 7.07	0 (0.00)		
Widowed	110	15.1	69.96 ± 12.19	3 (2.73)		
Unmarried	2	0.3	71.00 ± 1.41	0 (0.00)		
**Educational level**						
Primary and below	492	67.7	70.89 ± 10.10	8 (1.63)	*F* = 92.529	<0.001
Junior high school	135	18.6	83.13 ± 11.19	18 (13.33)		
High school/vocational high school/secondary school	81	11.1	84.38 ± 11.04	11 (13.58)		
University and above	19	2.6	93.26 ± 13.32	9 (47.37)		
**Previous occupation**						
Office	74	10.2	86.23 ± 13.33	16 (21.62)	*F* = 40.312	<0.001
Industrial and mining enterprises	88	12.1	75.94 ± 12.02	5 (5.68)		
Agricultural production activities	441	60.7	71.14 ± 9.70	9 (2.04)		
Commercial\attendant	65	8.9	82.15 ± 13.01	7 (10.77)		
Retiree	53	7.3	82.53 ± 12.29	6 (11.32)		
Other	6	0.8	92.67 ± 11.81	3 (50.00)		
**Family size (person)**						
1–2	342	47.0	77.30 ± 11.92	28 (8.19)	*F* = 9.199	<0.001
3–5	356	49.0	73.53 ± 12.44	16 (4.49)		
≥6	29	4.0	72.31 ± 12.45	2 (6.90)		
**Monthly per capita household income (Yuan)**						
<1,000	110	15.1	70.91 ± 11.69	4 (3.64)	*F* = 38.449	<0.001
1,000–2,999	409	56.3	73.36 ± 11.05	15 (3.67)		
3,000–4,999	188	25.9	79.82 ± 11.98	19 (10.11)		
≥5,000	20	2.8	94.90 ± 13.92	8 (40.00)		
**Smoking status**						
Never	61	8.4	79.57 ± 10.38	8 (13.11)	*t* = −2.874	0.004
Current	666	91.6	74.86 ± 12.43	41 (6.16)		
**Alcohol consumption**						
Never	42	5.8	79.64 ± 10.52	3 (7.14)	*t* = −2.385	0.017
Current	685	94.2	74.98 ± 12.39	43 (6.28)		

^a^Number of persons [composition (%)].

### 3.3 Correlation analysis of health literacy, social support, and health-related quality of life scores of older adults in combined medical and nursing care institutions

The results of Pearson's correlation analyses showed that the total health literacy scores, the total social support scores, and the total health-related quality of life scores of older adults in combined medical and nursing care institutions were positively correlated (*P* < 0.05). See Table 3 for details.

**Table 3:** Correlation analysis of health literacy, social support, and health-related quality of life scores of older adults in combined medical and nursing care institutions.

**Variable**	**Health literacy**	**Social support**	**Health-related quality of life**
Health literacy	1	0.134^**^	0.350^**^
Social support	0.134^**^	1	0.138^**^
Health-related quality of life	0.350^**^	0.138^**^	1

^**^At the 0.01 level (two-tailed), the correlation is significant.

### 3.4 Multiple linear regression analysis of the factors influencing health literacy of older adults in combined medical and nursing care institutions

The total health literacy score of older adults in combined medical and nursing care institutions was used as the dependent variable, and the statistically significant variables in the univariate and correlation analyses were used as the independent variables in the multiple linear stepwise regression analyses. The results showed that age, educational level, previous occupation, family size, monthly per capita household income, and health-related quality of life influenced health literacy among older adults in combined medical and nursing care institutions, which explained 40.2% of the total variation. The assignments of the independent variables are shown in Table 4, and the results of the linear regression analyses are shown in Table 5.

**Table 4:** Assignment of independent variables.

**Independent variable**	**Assignment method**
Social support rating total scale	Continuous variables, direct inclusion
Health-related quality of life total scales	Continuous variables, direct inclusion
Sex	Male = 1, female = 2
Smoking status	Current = 0, never = 1
Alcohol consumption	Current = 0, never = 1
Age (years)	60–69 (reference group) = 0000; 70–79 = 0100; 80–89 = 0010; ≥90 = 0001
Marital status	Married (reference group) = 0000; divorcee = 0100; widowed = 0010; unmarried = 0001
Educational level	Primary and below (reference group) = 0000; junior high school = 0100; high school/vocational high school/secondary school = 0010; university and above = 0001
Previous occupation	Office (reference group) = 000000; industrial and mining enterprises = 010000; agricultural production activities = 001000; commercial\attendant = 000100; retiree = 000010; other = 000001
Family size (person)	1–2 (reference group) = 000; 3–5 = 010; ≥6 = 001
Monthly per capita household income (Yuan)	<1,000 (reference group) = 0000; 1,000–2,999 = 0100; 3,000–4,999 = 0010; ≥5,000 = 0001

**Table 5:** Multiple linear regression analysis of the factors influencing health literacy of older adults in combined medical and nursing care institutions.

**Variable**	**β**	**SE**	**β^′^**	***t***	***P*-value**	**95.0% CI**
(Constant)	68.413	3.378		20.254	0.000	61.781 to 75.044
**Age (years)**						
70–79	−1.555	0.802	−0.063	−1.939	0.053	−3.129 to 0.020
80–89	−5.224	1.083	−0.164	−4.823	0.000	−7.350 to −3.098
≥90	−8.397	2.993	−0.079	−2.805	0.005	−14.273 to 2.520
**Marital status**						
Divorcee	1.643	6.617	0.007	0.248	0.804	−11.349 to 14.636
Widowed	−1.423	1.106	–0.041	−1.287	0.198	−3.594 to 0.748
Unmarried	2.222	6.486	0.009	0.343	0.732	−10.512 to 14.956
**Educational level**						
Junior high school	7.697	0.979	0.243	7.859	0.000	5.774 to 9.620
High school/vocational high school/secondary school	8.226	1.313	0.210	6.263	0.000	5.647 to 10.805
University and above	14.439	2.621	0.187	5.509	0.000	9.294 to 19.585
**Previous occupation**						
Industrial and mining enterprises	−5.458	1.587	−0.144	−3.440	0.001	−8.573 to −2.343
Agricultural production activities	−6.538	1.441	−0.259	−4.537	0.000	−9.367 to −3.709
Commercial\attendant	−0.726	1.723	−0.017	−0.421	0.674	−4.109 to 2.657
Retiree	−0.815	1.703	−0.017	−0.478	0.633	−4.158 to 2.529
Other	4.397	3.948	0.032	1.114	0.266	−3.355 to 12.149
**Family size (person)**						
3–5	−2.165	0.726	−0.088	−2.983	0.003	−3.590 to −0.740
≥6	−1.839	1.780	−0.029	−1.033	0.302	−5.334 to 1.657
**Monthly per capita household income (Yuan)**						
1,000–2,999	2.107	0.997	0.085	2.113	0.035	0.149 to 4.065
3,000–4,999	3.756	1.214	0.133	3.094	0.002	1.373 to 6.140
≥5,000	9.707	2.537	0.129	3.826	0.000	4.725 to 14.689
Sex	−0.300	0.740	−0.012	–0.406	0.685	−1.752 to 1.152
Smoking status	0.455	1.404	0.010	0.324	0.746	−2.302 to 3.212
Alcohol consumption	2.586	1.655	0.049	1.562	0.119	−0.664 to 5.835
Social support rating total scale	−0.049	0.064	−0.024	−0.764	0.445	−0.173 to 0.076
Health-related quality of life total scales	0.209	0.023	0.263	9.142	0.000	0.165 to 0.254

R^2^ = 0.420, adjusted R^2^ = 0.402, F = 23.161, P <0.001.

## 4 Discussion

### 4.1 Analysis of the health literacy of older adults in combined medical and nursing care institutions

In this study, the health literacy score of older adults in combined medical and nursing care institutions was 75.25 ± 12.33, which was higher than those of older adults in Yinchuan City, China (30) and Urumqi City, China (19). The reasons may be related to geographical differences, the level of economic development, and older adult's use of health-related resources. Only 6.33% of the older adults in this study had basic health literacy, and the scores for the dimensions of health literacy were, in descending order, ability to communicate and interact, ability to access information, willingness to improve health, and willingness to support financially. Although older adults had a certain degree of health information acquisition, communication, and interaction, there may have been cognitive and economic problems, resulting in a particular gap in transforming health knowledge into health behavior. Therefore, we recommend that the relevant staff of nursing care institutions work together with medical institutions to conduct health education and promotion activities, popularize health knowledge. Furthermore, they should disseminate health knowledge and skills through scientific and authoritative channels, enhance the grasp and understanding of health knowledge among older adults, and help older adults scientifically recognize health problems and implement health behaviors so that they will be willing to improve their existing health problems and enhance their health literacy.

### 4.2 Analysis of factors influencing the health literacy of older adults in combined medical and nursing care institutions

#### 4.2.1 Age

The results of this study showed that the health literacy level of older adults 80–89 years of age vs. those aged ≥90 years of age was lower than that of younger adults 60–69 years of age. Consistent with the findings of Fry et al. (31), with age, the cognitive level of older adults gradually declined, and comprehension decreased, leading to a decrease in the ability of older adults to acquire and master health information and, thus, a decrease in health literacy. It is recommended that when health education is conducted for older adults of different ages, the distribution of brochures, the broadcasting of videos, and the use of a variety of methods in health lectures be chosen to disseminate health-related information in an easy-to-understand manner. Furthermore, health education activities should be conducted to improve the level of health literacy among older adults of advanced ages.

#### 4.2.2 Educational level

This study showed that as the educational level increased, the health literacy level of older adults increased, which is consistent with the results of previous studies (13, 32). The higher the educational level, and the better the knowledge base of older adults, the better their ability and initiative in acquiring, processing, and understanding information, and the stronger their sense of self-care, the more they will take the initiative to obtain various health information. In contrast, older adults with lower educational levels are slower to accept new things, have poorer self-understanding, have limited awareness of disease prevention, and find it challenging to acquire scientific and adequate health knowledge and skills, resulting in lower health literacy (33, 34). In this survey, the literacy level of older adults was usually low, with 67.7% of older adults having a literacy level of primary and below. These results suggest that the relevant local authorities should pay attention to older adults with a lower literacy level, and conduct long-term corresponding health education for older adults at different literacy levels. Methods such as the teach-back method should be used to promote older adults' understanding of the relevant health information, to improve their health literacy level.

#### 4.2.3 Previous occupation

This study showed that older adults who had worked in industrial and mining enterprises and those who had been engaged in agricultural production activities had lower levels of health literacy than those who had worked in offices. Work in industrial and mining enterprises and agricultural production activities involves manual labor, while work in an office involves mental activities. Previous studies (35, 36) have reported that occupation as a manual laborer is a risk factor for the health literacy of older adults in China. The health literacy level of manual laborers is significantly lower than that of office worker laborers, which may be related to the fact that manual laborers have fewer chances to receive health-related training than those who are office workers, as they become older adults (37, 38). Their income level decreases, and they focus more on sustaining their livelihoods, ignoring their physical health, which, in turn, influences the status of their health literacy. Therefore, the relevant government agencies should pay attention to older adults engaged in manual work, increase publicity on health education, and conduct health promotion and health science activities through multiple channels and in multiple ways to improve their health literacy.

#### 4.2.4 Family size

This study showed that older adults with a family size of 3–5 members were less health literate than those with a family size of 1–2 members. Consistent with the results of a study in Jiangsu Province, China (39), the reason for this may involve the socio-cultural context of China; older adults tend to live with their children or grandchildren. As the number of family members increases, their attention is more focused on their children or grandchildren (40). They pay less attention to their health, and their health literacy needs to be addressed. Therefore, volunteers are encouraged to enter nursing care institutions and interact with older adults, to increase their health knowledge, learn health skills, and improve their health literacy.

#### 4.2.5 Monthly per capita household income

This study showed that the health literacy level of older adults increased as the monthly per capita household income increased, which is consistent with the findings of Kim (41) involving older adults in Korea. The higher the monthly per capita household income, the better the economic base, the more social resources they can obtain, the better the health-related quality of life, the more attention they will pay to their health status, the more health information they will obtain, and thus the better the level of health literacy (42, 43). The relevant departments are therefore encouraged to pay more attention to the health literacy of older adults with lower monthly per capita household income, to improve the ability of older adults with financial difficulties to obtain and understand health-related information, and to enhance their health literacy levels.

#### 4.2.6 Health-related quality of life

This study showed that health-related quality of life considerably influences the health literacy of older adults in nursing care institutions. We found that the higher the health-related quality of life of older adults in nursing care institutions, the higher the level of health literacy. This finding is consistent with the results of the study by Liu et al. (17). Older adults who perceived themselves to be in better health and were more health-conscious had a greater awareness of physical healthcare and health information needs, and were more likely to seek health knowledge and skills and practice them in their daily lives to promote healthy lifestyles and behavior and improve health-related quality of life (44, 45). Therefore, healthcare providers should identify people with low levels of health literacy and develop programs for different health conditions based on individual circumstances. They should also reduce jargon in communication to promote their patients' understanding of relevant health knowledge to improve the health-related quality of life and health outcomes.

### 4.3 Correlation analysis of health literacy, social support, and health-related quality of life

The results of this study showed that the health literacy of older adults in combined medical and nursing care institutions was positively correlated with social support (*P* < 0.05), suggesting that the higher the level of social support, the higher the level of health literacy. Some studies have reported that social support had an essential influence on health literacy and overall health status, and that higher levels of social support positively influenced health literacy and health status (46, 47). In our study, health literacy was positively associated with the health-related quality of life (*P* < 0.05), similar to the findings of Zheng et al. (48). Multiple linear stepwise regression results also showed that the higher the health-related quality of life, the higher the relative level of health literacy among older adults. Older adults with lower levels of health literacy may need more access to healthcare resources. They may need to more clearly articulate their needs when communicating with professionals, such as doctors, to seek additional healthcare services, ultimately leading to increased health-related quality of life (49). Therefore, to improve the health literacy of older adults, the positive impact of social support and health-related quality of life should be emphasized. When faced with older adults with a low level of health literacy, workers should patiently communicate in an easy-to-understand manner, guiding them to use the social support system they already have to improve their health and health literacy.

## 5 Limitations

This study had some limitations. First, in the selection of the research subjects, this study only investigated some of the older adults in combined medical and nursing care institutions in Chengdu City, China, which could only partially reflect the actual health literacy level of all older adults in China. Because health literacy levels are closely related to the level of regional economic development, it is therefore necessary to include a broader range of older adults in future studies so that the results of this study can be based on a representative sample. Second, in terms of research methodology, this study conducted a cross-sectional survey using a questionnaire survey method without any interventions and control of relevant factors, so subsequent prospective cohort studies should be conducted on the basis of this study to further determine effective measures to improve the health literacy of older adults in combined medical and nursing care institutions.

## 6 Summary

To summarize, the health literacy level of older adults in combined medical and nursing care institutions was low, fewer older adults have basic health literacy, and the health outlook could be improved. Combined medical and nursing care institutions and relevant departments should pay closer attention to factors affecting the health literacy of older adults. Furthermore, they should help older adults correctly understand aging, establish a positive outlook on aging, and conduct health education activities for older adults using online and offline methods (such as conducting health education bulletin boards, broadcasting health education promotional videos, and holding health clinics, etc.). Doing so will promote the enhancement of health literacy in the form of a tripartite linkage between the government, society, and institutions to better realize positive and healthy aging.

## Data availability statement

The original contributions presented in the study are included in the article/supplementary material, further inquiries can be directed to the corresponding author.

## Author contributions

ST: Conceptualization, Writing – original draft. SS: Conceptualization, Writing – original draft. SW: Methodology, Writing – review & editing. TP: Formal analysis, Writing – review & editing. LC: Methodology, Writing – review & editing. MY: Investigation, Writing – original draft. JM: Investigation, Writing – original draft. HL: Conceptualization, Validation, Writing – review & editing.
